# A temperature responsive hydrogel encapsulated with adipose‐derived stem cells and melanin promotes repair and regeneration of endometrial injury

**DOI:** 10.1002/btm2.10714

**Published:** 2024-08-16

**Authors:** Ruigao Song, Chicheng Ma, Hongxia Li, Yu Cheng, Xianmei Cui, Zanhong Wang, Lijuan Huang, Chunying Song, Yukai Jing, Bing Cao, Lili Wang, Qing Tian, Xi Wang, Ruiping Zhang, Hanwang Zhang

**Affiliations:** ^1^ Shanxi Bethune Hospital, Shanxi Academy of Medical Sciences, Third Hospital of Shanxi Medical University, Tongji Shanxi Hospital Taiyuan China; ^2^ College of Animal Science, Shanxi Agricultural University Taigu China; ^3^ The Radiology Department of Shanxi Provincial People's Hospital The Fifth Hospital of Shanxi Medical University Taiyuan China; ^4^ Tongji Hospital, Tongji Medical College, Huazhong University of Science and Technology Wuhan China

**Keywords:** ADSCs, endometrium, hydrogel, MNP, regeneration

## Abstract

The endometrium, the inner lining of the uterus, assumes a crucial role in the female reproductive system. Disorders and injuries impacting the endometrium can lead to profound consequences, including infertility and compromised women's overall health. Recent advancements in stem cell research have opened new possibilities for the treatment and repair of endometrial issues. In the present study, we constructed a degradable hydrogel by loading adipose‐derived stem cells (ADSCs) and melanin nanoparticles (MNP). In vitro cell experiments validated the biocompatibility of the prepared hydrogels and their adeptness in encapsulating ADSCs. Subsequently, we explored the impact of hydrogel@ADSC@MNP constructs in the healing process of uterine injury in mice. The results indicated that hydrogel@ADSC@MNP could augment endometrial thickness and ameliorate endometrial interstitial fibrosis. The injured tissue adjacent to hydrogel@ADSC@MNP constructs exhibited higher levels of bFGF, IGF‐1, and VEGFA compared with the corresponding tissue in mice receiving hydrogel constructs alone or in the model group. Furthermore, the hydrogel@ADSC@MNP system enhanced the proliferative capabilities of uterine endometrial cells, facilitated microvasculature regeneration, and reinstated the endometrium's capacity to receive the embryos. Our findings strongly suggest that the hydrogel@ADSC@MNP system holds significant promise for repairing and regenerating damaged endometrium.

## 1 INTRODUCTION

The endometrium is a unique tissue within the female reproductive system that undergoes cyclic processes of growth, differentiation, shedding, and renewal in each menstrual cycle. Successful embryo implantation in the uterus relies on high‐quality embryos and a receptive endometrial environment. This remarkable ability for regeneration and transformation is essential for women's reproductive health. Despite advancements in assisted reproductive technologies leading to improved fertilization rates and enhanced embryo culture conditions, implantation remains a bottleneck for the success of in vitro fertilization‐embryo transfer (IVF‐ET).^1^ However, severe damage to the endometrium, such as dilation and curettage, endometrial inflammation, myomectomy, or endometrial tuberculosis, can lead to thinning of the endometrium, intrauterine adhesions, and endometrial scarring. These issues can further lead to abnormal uterine bleeding, miscarriages, pregnancy complications, or infertility.^2^, ^3^

Several strategies have been explored for the treatment of endometrial damage. To improve endometrial thickness, a range of therapeutic methods has been investigated, including the use of growth factors, exogenous estrogens, pentoxifylline, sildenafil, vitamin E, and low‐dose aspirin, among others. However, these approaches have not demonstrated significant clinical efficacy.^4^, ^5^, ^6^ In the context of addressing intrauterine adhesions, hysteroscopy is commonly used as the primary diagnostic tool and the most frequently used treatment method.^7^ Despite the application of other strategies to restrict or prevent re‐adhesions, postoperative recurrence rates remain notably high, with moderate adhesions occurring in 23% of cases and severe adhesions in 62%.^8^ Meanwhile, there are limited methods available for effectively addressing severe scarring of the endometrium following surgical procedures.^9^, ^10^, ^11^

Consequently, there is an urgent need for more effective treatments aimed at repairing damaged endometrial structure, improving endometrial receptivity, and enhancing embryo implantation rates. Stem cell therapy has emerged as a potential method to address these challenges by harnessing the regenerative power of stem cells to repair and restore the endometrial tissue. Mesenchymal stem cells (MSCs) are characterized by their remarkable potential for proliferation and differentiation into various cell types.^12^ Currently, there have been reports of research on the utilization of MSCs for the regeneration and repair of the endometrium.^13^, ^14^ In endometrial injuries, fibrosis plays a crucial role in adhesions and severe scarring within the uterine cavity. The potential of MSCs in treating fibrosis has been widely confirmed in other tissues and organs. In animal models of pulmonary fibrosis,^15^ liver fibrosis,^16^ and renal fibrosis,^17^ MSCs have been shown to reduce fibrosis. Among MSCs, adipose‐derived stem cells (ADSCs) represent a subset of adult stem cells distinguished by their inherent attributes, including self‐renewal capability, pluripotent differentiation potential, immunomodulatory properties, and low immunogenicity. Moreover, ADSCs exhibit an additional advantageous trait: the secretion of trophic factors, which substantially contribute to the enhancement of therapeutic and regenerative effects across a diverse spectrum of applications. It is noteworthy that, in the context of regenerative medicine, ADSCs have emerged as the most auspicious and highly‐regarded cellular resource.^18^

Traditionally, stem cell therapy involves direct injection into the damaged tissue, but this method has limited efficiency in delivering cells to the target. Natural extracellular matrices (ECMs) have been extensively used for ADSCs adhesion, migration, differentiation, and proliferation studies. However, the inferior mechanical properties and unpredictable biodegradation characteristics of natural ECMs limit their biomedical feasibility, increasing the demand for different synthetic scaffolds. Hydrogels, due to their excellent swelling properties and similarity to soft tissues, are considered the most promising alternative materials.^18^

Melanin nanoparticle (MNP), an easily obtainable and cost‐effective biopolymer, offers biocompatibility, biodegradability, and metal chelating abilities. This biomaterial can serve as a nanocarrier in biomedical field, with applications in nano‐imaging, drug‐controlled release, and bioengineering.^19^, ^20^, ^21^, ^22^ Furthermore, MNP is a low‐toxicity novel antioxidant and anti‐inflammatory drug. It can protect against γ‐ray induced DNA damage in mice and significantly reduce the serum IL‐1β content in a rat model of non‐alcoholic fatty liver disease, while also restoring the levels of cytokines IL‐10 and TGF‐β to normal.^23^, ^24^

**FIGURE 1:**
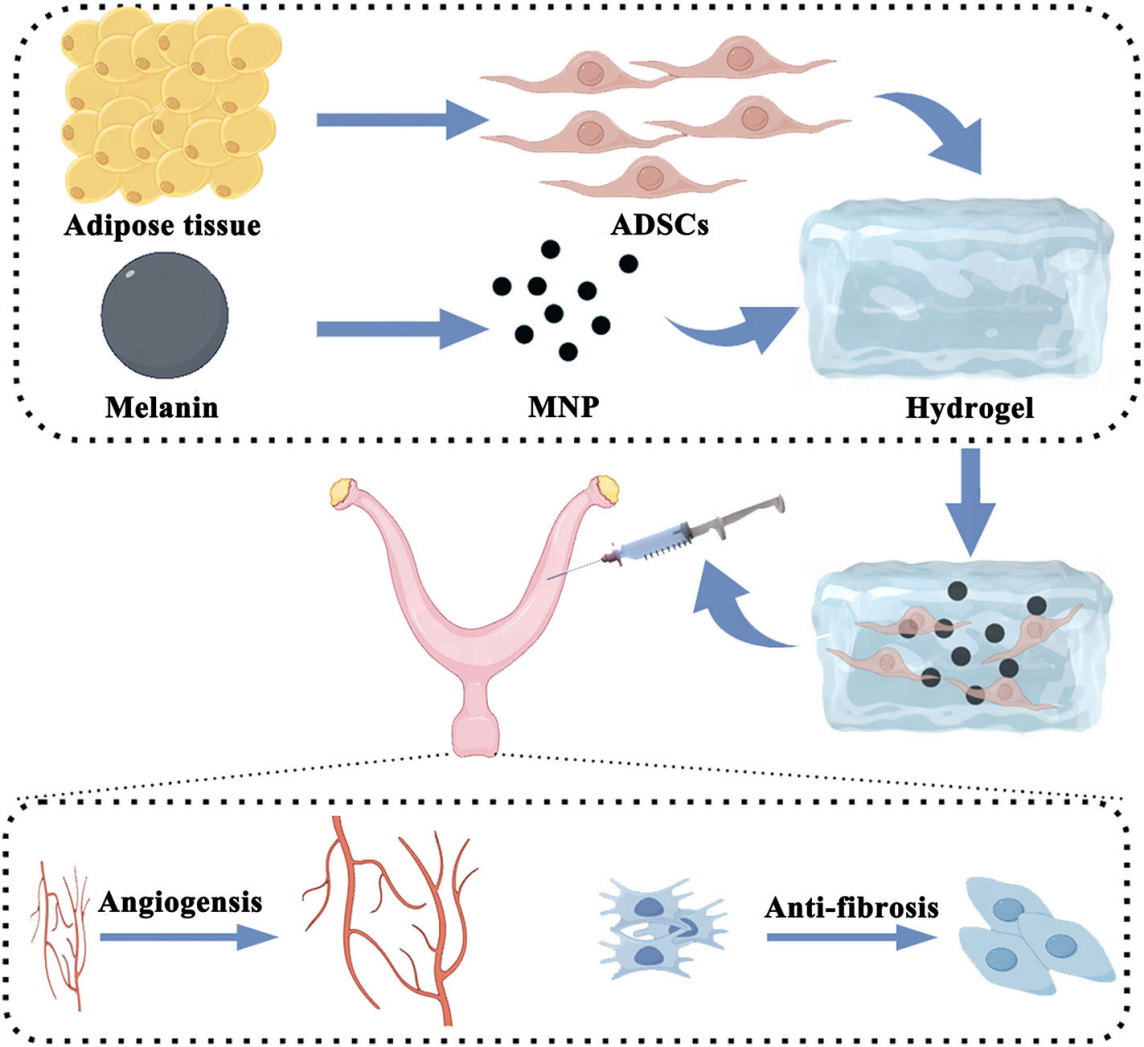
Schematic overview of the development of a hydrogel encapsulated with ADSCs and MNP for endometrial regeneration, depicted by Figdraw. The ensuring hydrogel@ADSCs@MNP was injected into the uterine cavity, wherein it mediated sustained ADSCs and MNP release to facilitate the regeneration of the endometrium, promoted neovascularization, and anti‐fibrotic activity.

Thus, in this study, we evaluated the therapeutic effects of the thermosensitive hydrogel coupled with MNP and cross‐linked with stem cells in endometrial injury mouse models (Figure 1). This composite material exhibits advantageous in promoting endometrial self‐healing and antioxidative properties, thereby manifesting substantial clinical applicability potential.

## 2 RESULTS

### 2.1 Characterization of ADSCs

**FIGURE 2:**
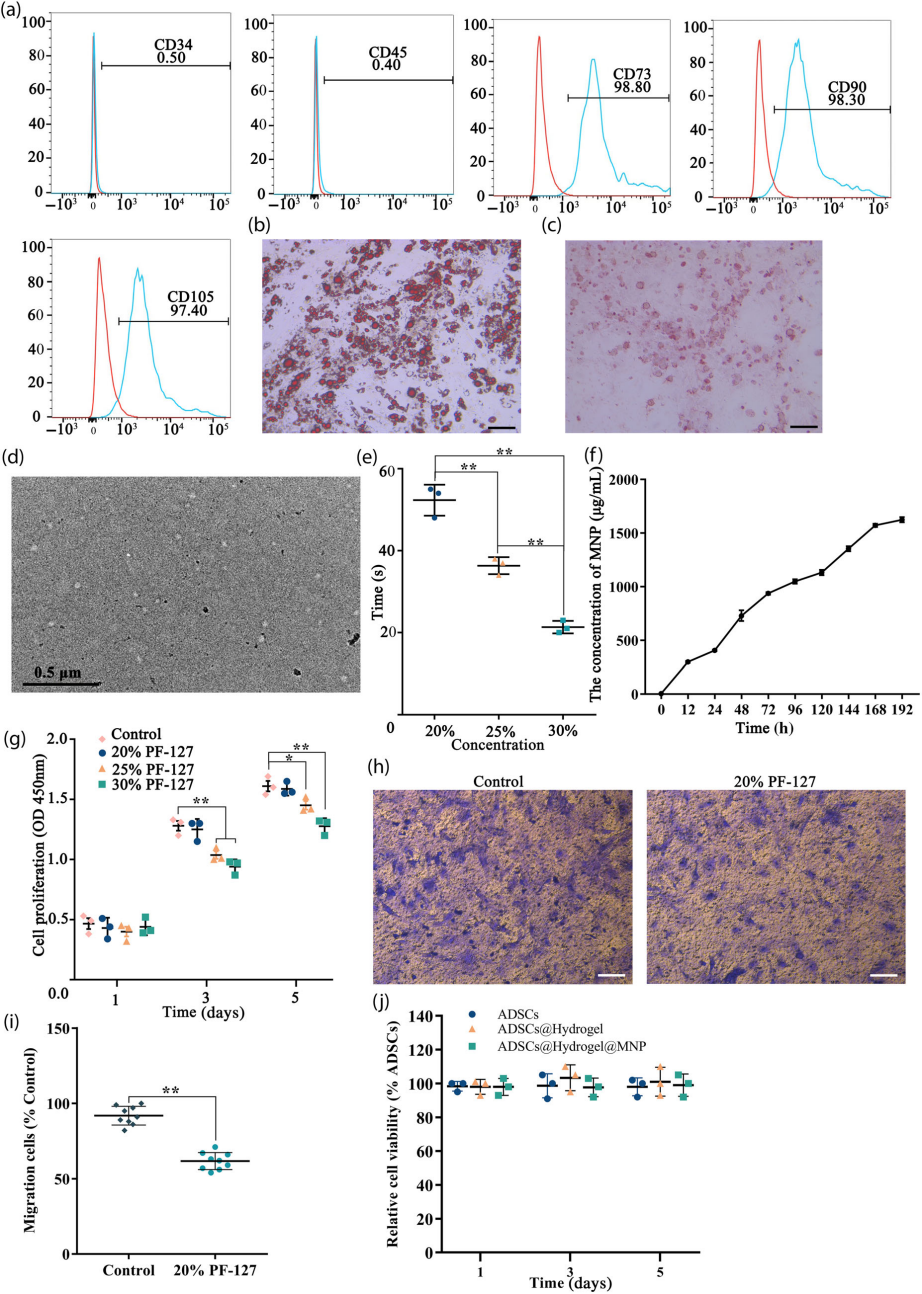
Characterization of adipose‐derived mesenchymal stem cells (ADSC) and in vitro biocompatibility assessment. (a) Flow cytometric analysis of ADSC. ADSC were positive for CD90 and CD105 and negative for CD34 and CD45. (b) Adipogenic differentiation was stained by Oil Red O (scale bar =100 μm). (c) Osteogenic differentiation was confirmed through Alizarin Red S staining (scale bar = 100 μm). (d) Characterization of MNP. (e) Assessment of gelation time at 37°C for various concentrations of hydrogel. (f) The concentration of melanin released from the hydrogel into the physiological saline at different time points. (g) Proliferation rates of ADSCs within hydrogels of different concentrations. The cell migration of ADSCs in a 20% hydrogel (h) and the statistical comparison with the control group (i). (j) The relative cell viability of ADSCs, ADSCs@Hydrogel and ADSCs@Hydrogel@MNP. *p < 0.05, **p < 0.01.

The cell surface antigen expression profile, as determined by flow cytometry analysis, indicated that ADSCs exhibited positive immunoreactivity for CD73, CD90, and CD105, negative immunoreactivity for CD34 and CD45 (Figure 2a). Adipogenic differentiation was confirmed through positive Oil Red O staining, highlighting the presence of lipid droplets within the induced ADSCs population (Figure 2b). Additionally, the differentiation potential of ADSCs into osteogenic lineage was substantiated by Alizarin red staining, which revealed the presence of a calcified extracellular matrix (Figure 2c). These findings concordant with the characteristic features of mesenchymal stem cells.

### 2.2 Synthesis and characterization of MNP

To enhance the inherent poor water solubility of melanin, pristine melanin granules were initially dissolved in 0.1 N NaOH and subsequently neutralized with the assistance of sonication to minimize interchain aggregation. Ultra‐small melanin nanoparticles (MNP) with high water monodispersity and homogeneity, measuring less than 10 nm in size, were successfully obtained, and termed as plain water‐soluble MNP (Figure 2d).

### 2.3 In vitro assessment of ADSCs and hydrogel biocompatibility

For the in vitro assessment of ADSCs and hydrogel biocompatibility, the encapsulation and cultivation of ADSCs within hydrogels were conducted. A higher concentration of PF‐127 results in a shorter gelation time at 37°C (Figure 2e). In addition, MNP undergoes a slow‐release process in the hydrogel (Figure 2f). CCK‐8 method was used to measure the proliferation of ADSCs in the hydrogel after 1, 3, and 5 days of incubation. Figure 2g illustrates that the optical density (OD) value of the 20% PF‐127 hydrogel encapsulated with ADSCs group did not show a significant difference compared with the control group. The Transwell results indicated that ADSCs could migrate out of the hydrogel, with migration speed significantly lower than that of the control group (Figure 2h,i). Additionally, cell viability was assessed for ADSCs, hydrogel@ADSC, and hydrogel@ADSC@MNP groups after 5 days of culture, revealing no significant differences among the three groups (Figure 2j). These results indicate excellent biocompatibility of MNP and hydrogel, promoting cell proliferation and maintaining cell viability.

### 2.4 Histological examination analysis

**FIGURE 3:**
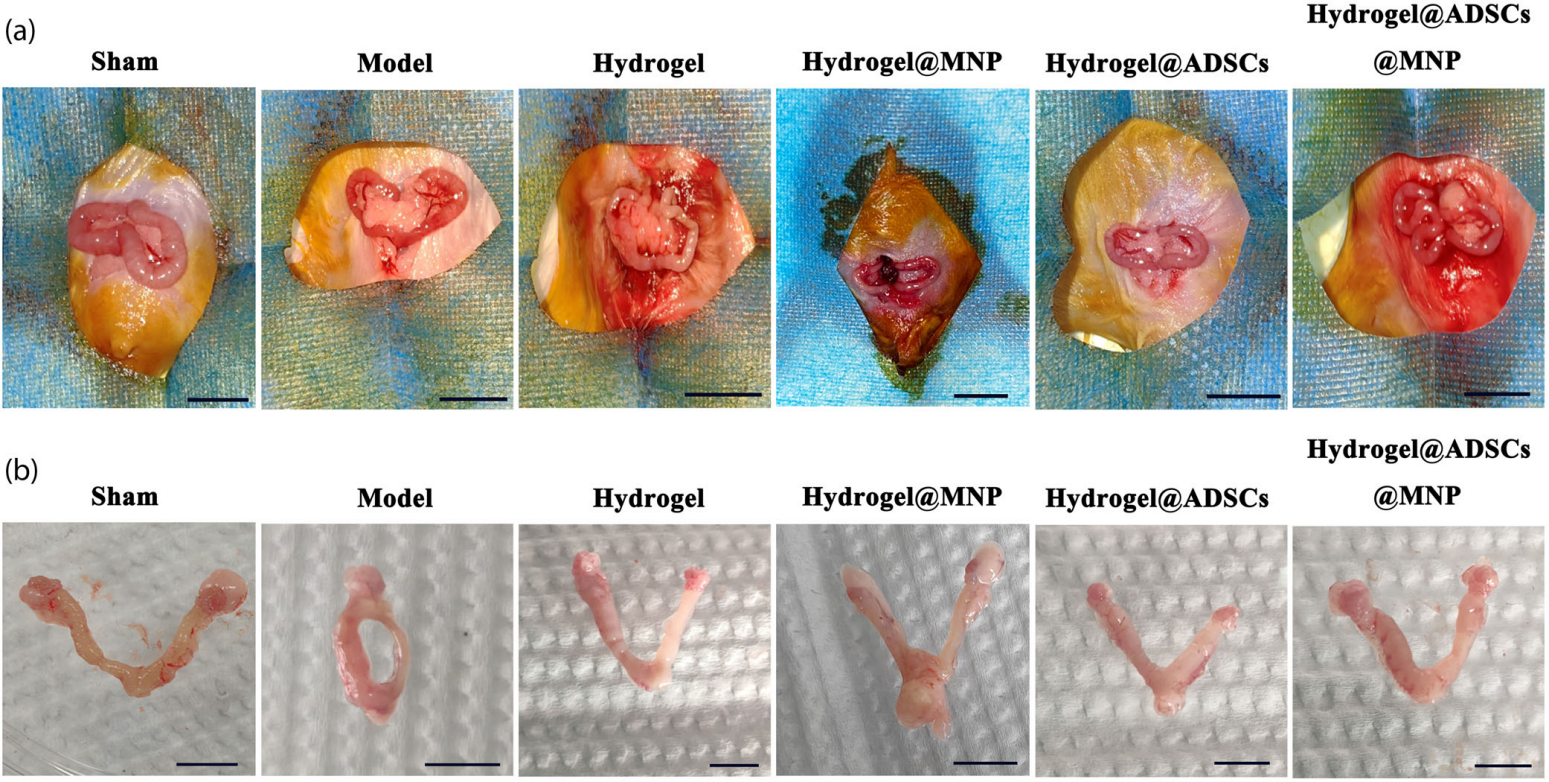
Establishment and treatments of a mice model of uterine injure. (a) After all mice were anesthetized by intraperitoneal injection of 60 mg/kg pentobarbital, a low abdominal midline incision was made to expose uterine horns. PBS, hydrogel, Hydrogel@MNP, Hydrogel@ADSCs or Hydrogel@ADSCs@MNP were injected into each right uterine (scale bar = 1 cm). (b) the mice were humanely euthanized, and their uterine tissues were excised at day 21 during the diestrus stage of three estrus cycles (scale bar = 0.5 cm).

Subsequently, we established a mouse model of endometrial injury by directly injecting 95% ethanol into the uterine horn. Following the modeling process, mice in both the model group and the sham operation group exhibited normal mental states, maintained a healthy diet, and experienced regular estrous cycles. During the modeling process, the uterine morphology of mice in all groups appeared normal (Figure 3a). After three estrous cycles, Figure 3b illustrates that the uterine morphology in the sham operation group and the hydrogel@ADSC@MNP group remained regular, uniform, and elastic. In contrast, the uteri in the model group exhibited inelastic shrinkage, adhesions, and viable necrotic tissue.

**FIGURE 4:**
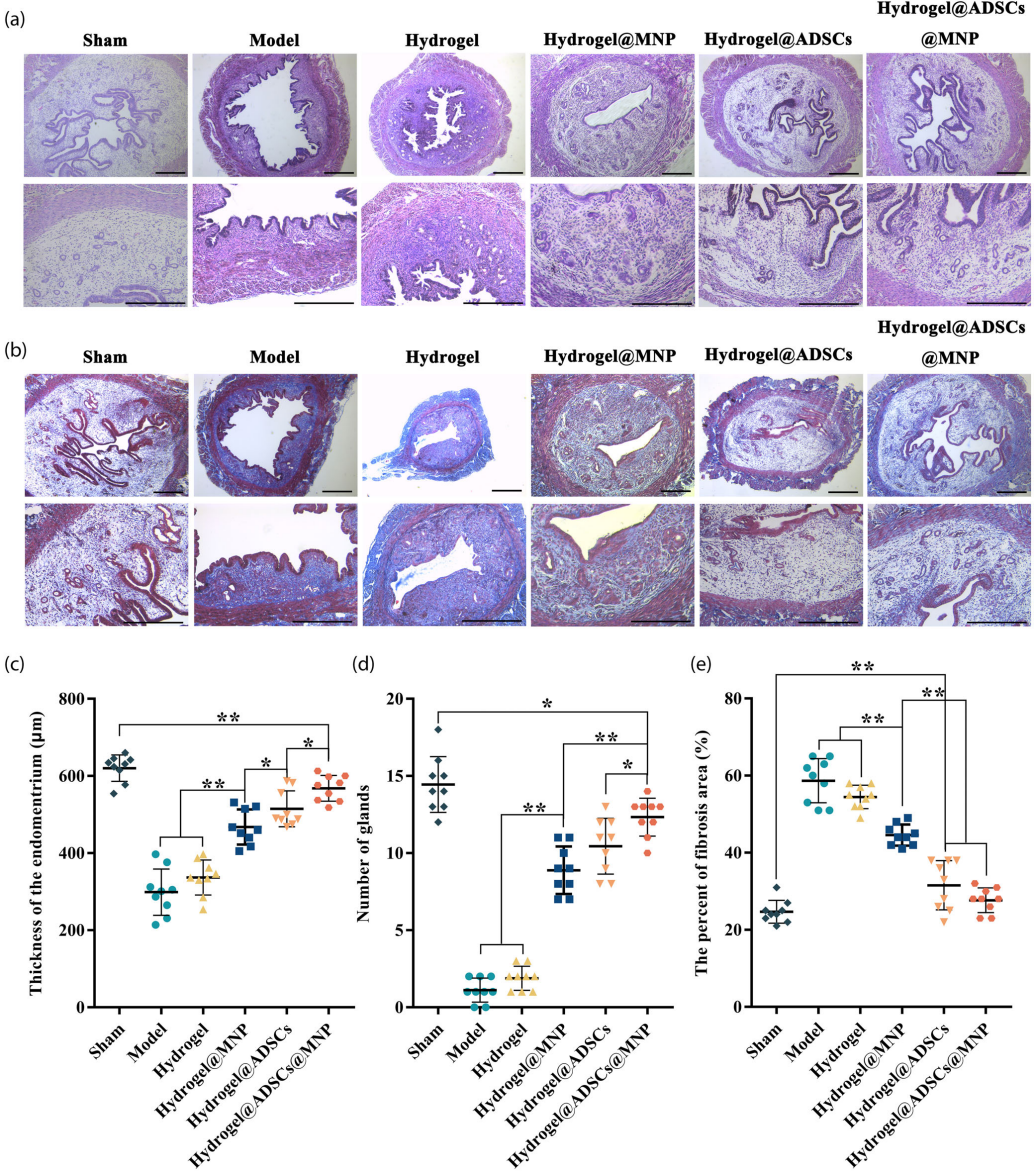
H&E and Masson staining of the uterine tissue in different groups. (a) H&E staining of the uterine tissue (scale bar = 400 μm); (b) Masson staining of the uterine tissue in different groups (scale bar = 400 μm); (c) Quantification of the endometrial thickness; (d) Quantification of the number of the glands; (e) Quantification of the fibrosis area percentage. Data are presented as mean ± SEM. *p < 0.05, **p < 0.01.

H&E staining revealed that the endometrial surface of the sham group was covered with simple high columnar epithelial cells, with tiny blood vessels and endometrial glands primarily located in the submucosa and basal layer (Figure 4a). Conversely, the uterine surface of the model group was covered by flat low columnar epithelial cells, and the glands beneath the epithelial layer were reduced. Following the administration of hydrogel@MNP, hydrogel@ADSC and hydrogel@ADSC@MNP, the endometrial surface reverted to high columnar epithelial cells. Maintaining an appropriate endometrial thickness is crucial for providing an optimal site for embryo implantation, a key factor for ensuring a successful pregnancy outcome. In the hydrogel@ADSC@MNP group, the thickness of the endometrium was found to be comparable to that of the sham operation group (Figure 4c). It is noteworthy that the endometrial thickness in the model group exhibited a significant decrease compared with the sham operation group.

Both the sham group and the hydrogel@ADSC@MNP treatment group demonstrated a significantly higher number of endometrial glands compared with the model group (Figure 4d). Masson's trichrome staining revealed that the uterine tissue in the model group exhibited a substantial presence of fibrillates (Figure 4b). In contrast, the fibrotic area in the hydrogel@ADSC@MNP treatment group displayed a reduced quantity of fibrillates, attributed to the inhibitory effect of ADSCs on endometrial fibrosis (Figure 4e).

### 2.5 Assessment of the in vivo angiogenic and regenerative activity

**FIGURE 5:**
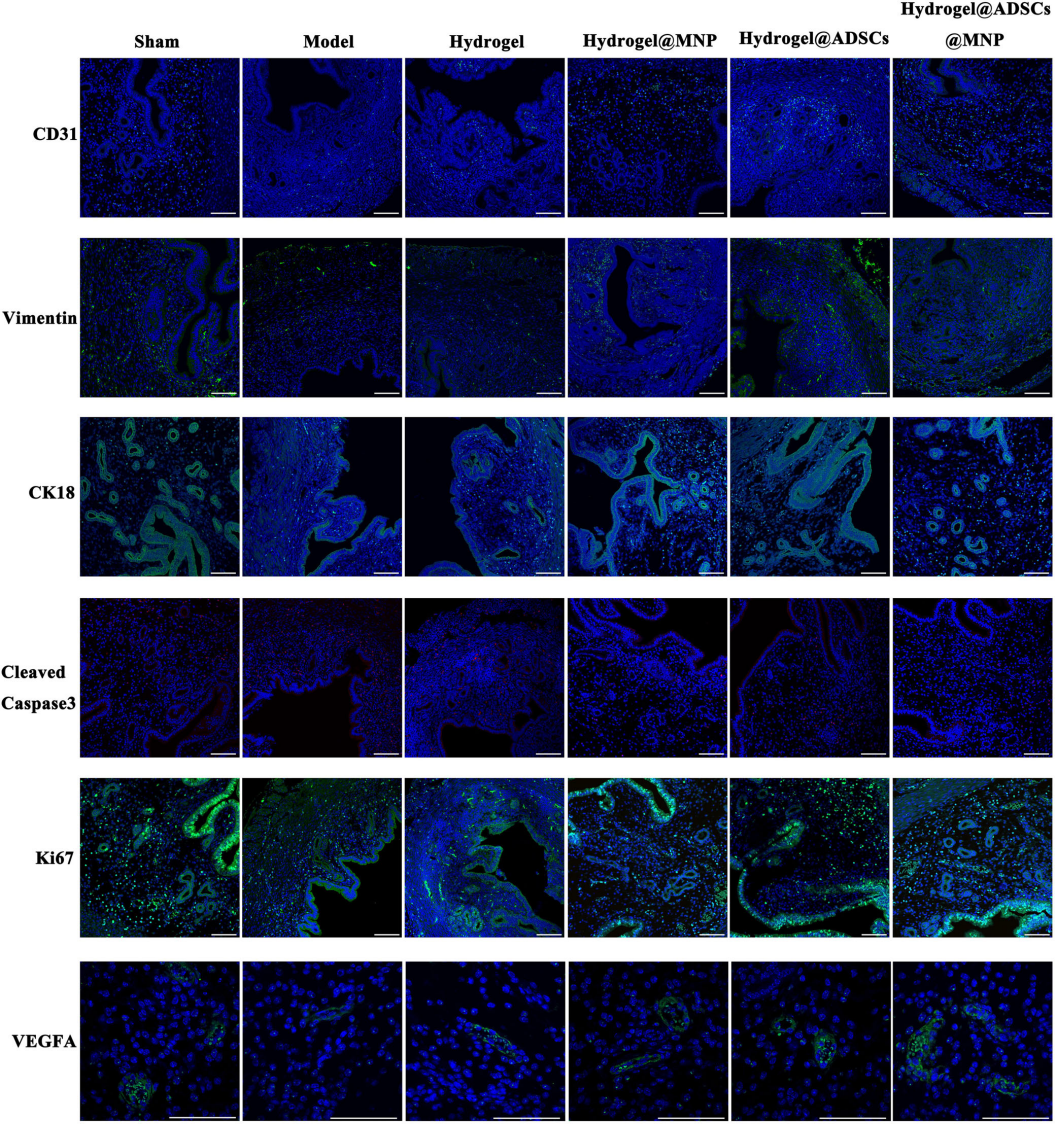
Representative CD31, Vimentin, CK18, Cleaved Caspase3, Ki67 and VEGFA images of immunofluorescence staining of uterine samples (scale bar = 100 μm).

**FIGURE 6:**
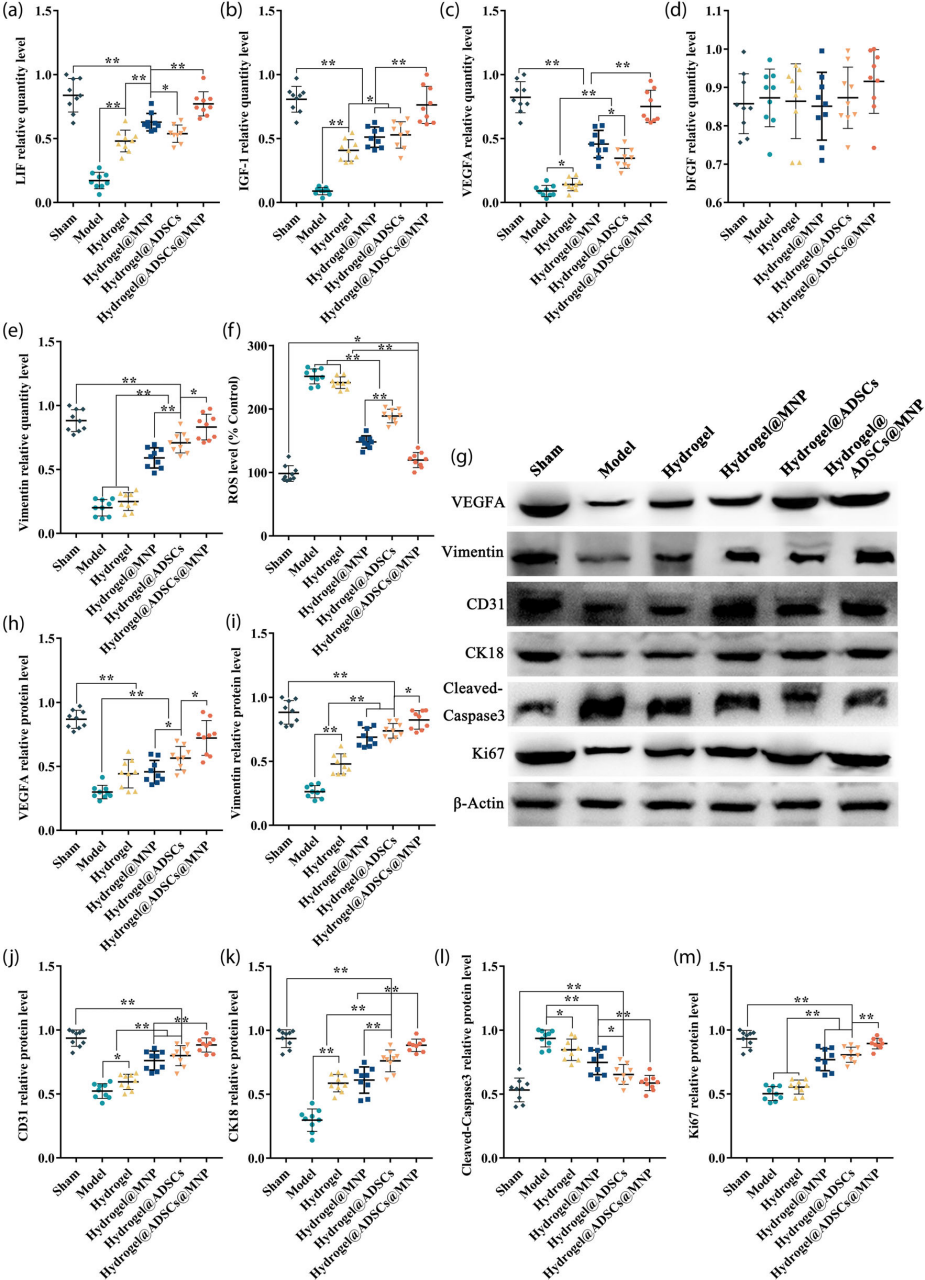
The impact of hydrogel@ADSCs@MNP transplantation on the expression of markers of endometrial receptivity and angiogenesis. (a–e) A qPCR approach was used to assess the expression of markers of endometrial receptivity and angiogenesis, with β‐Actin serving as a normalization control. (f) The ROS level of different treatment groups. (g) Western blotting was used to assess the protein expression of VEGFA, Vimentin, CD31, CK18, Cleaved Caspase3, and Ki67 in each treatment group. H‐M) Western blotting data depicting the levels of VEGFA, Vimentin, CD31, CK18, Cleaved Caspase3, and Ki67 in various treatment groups. The data are presented relative to internal reference controls. Data are means ± standard error, *n* = 9. *p < 0.05, **p < 0.01.

In the subsequent analysis, we assessed the expression levels of key markers associated with endometrial angiogenic and regenerative, including Differentiation 31 (CD31), VEGFA, Vimentin, cytokeratin 18 (CK18), cell proliferation antigen Ki‐67 (Ki67) and Cleaved Caspase 3. These markers play pivotal roles in various developmental processes. Illustrated in Figure 5, the distribution of CD31 in the sham operation group surpassed that in the model group. In contrast, the CD31 expression in the hydrogel@ADSC@MNP treatment group showed no difference compared with that in the sham operation group. Additionally, the vascular structures represented by VEGFA positivity were significantly reduced in the model group, whereas in the hydrogel@ADSCs@MNP treatment group, the expression level was markedly increased compared with the model group (Figure 5). CK18, a member of the keratin family of intermediate filament proteins, exhibited heightened expression in the cytoplasm of endometrial epithelial cells. As depicted in Figure 5, CK18 expression in the sham operation group exceeded that in the model group. Additionally, both the hydrogel@ADSC treatment group and the hydrogel@ADSC@MNP treatment group demonstrated elevated CK18 expression compared with the model group. Vimentin, indicative of specific expression in endometrial stromal cells, exhibited variations in different treatment groups. Figure 5 shows that Vimentin expression in the sham operation group exceeded that in the model group. The hydrogel@ADSC@MNP treatment group exhibited increased Vimentin expression, mirroring the levels observed in the sham group. This observation suggests that the amalgamation of hydrogels with ADSC and MNP may yield a more efficacious therapeutic outcome. The cell proliferation marker Ki67 served as a crucial index for assessing cellular proliferation. Figure 5 illustrates that the expression of Ki67 in the hydrogel@ADSC@MNP treatment group surpassed that in the model group, suggesting enhanced cell proliferation and tissue growth. Caspase‐3 activation is a distinctive feature of apoptotic cell death. As depicted in Figure 5, the expression of Cleaved‐Caspase 3 in both the hydrogel@ADSC treatment group and the hydrogel@ADSC@MNP treatment group is significantly lower than that observed in the model group. This trend is further supported by western blot analysis, as illustrated in Figure 6g–m. Notably, protein expression levels of CD31, CK18, Vimentin and VEGFA in the hydrogel@ADSC@MNP group are significantly elevated compared with the model group, approaching levels observed in the sham group.

The assessment of oxidative stress in uterine tissues was conducted using the ROS fluorescence probe 2′, 7′‐dichlorofluorescin diacetate (DCFH‐DA) (Figure 6f). In comparison with the sham group, the model group exhibited a significant increase in ROS levels. Conversely, the hydrogel@ADSC@MNP group demonstrated ROS levels nearly identical to those of the sham group, suggesting superior ROS scavenging ability in this treatment group.

Subsequently, we evaluated the expression of LIF and IGF‐1, recognized markers of endometrial receptivity with pleiotropic effects on various associated developmental processes. Additionally, the expression of VEGFA and bFGF, known for promoting neovascularization and endothelial cell proliferation, were also assessed. At the mRNA level, the hydrogel@ADSC treatment resulted in a significant upregulation of LIF, IGF‐1 and VEGFA expression (Figure 6a–e). Importantly, the hydrogel@ADSC@MNP treatment group exhibited a further increase in the expression of these markers.

### 2.6 Assessment of the ability of hydrogel@ADSC@MNP to promote pregnancy outcomes

**FIGURE 7:**
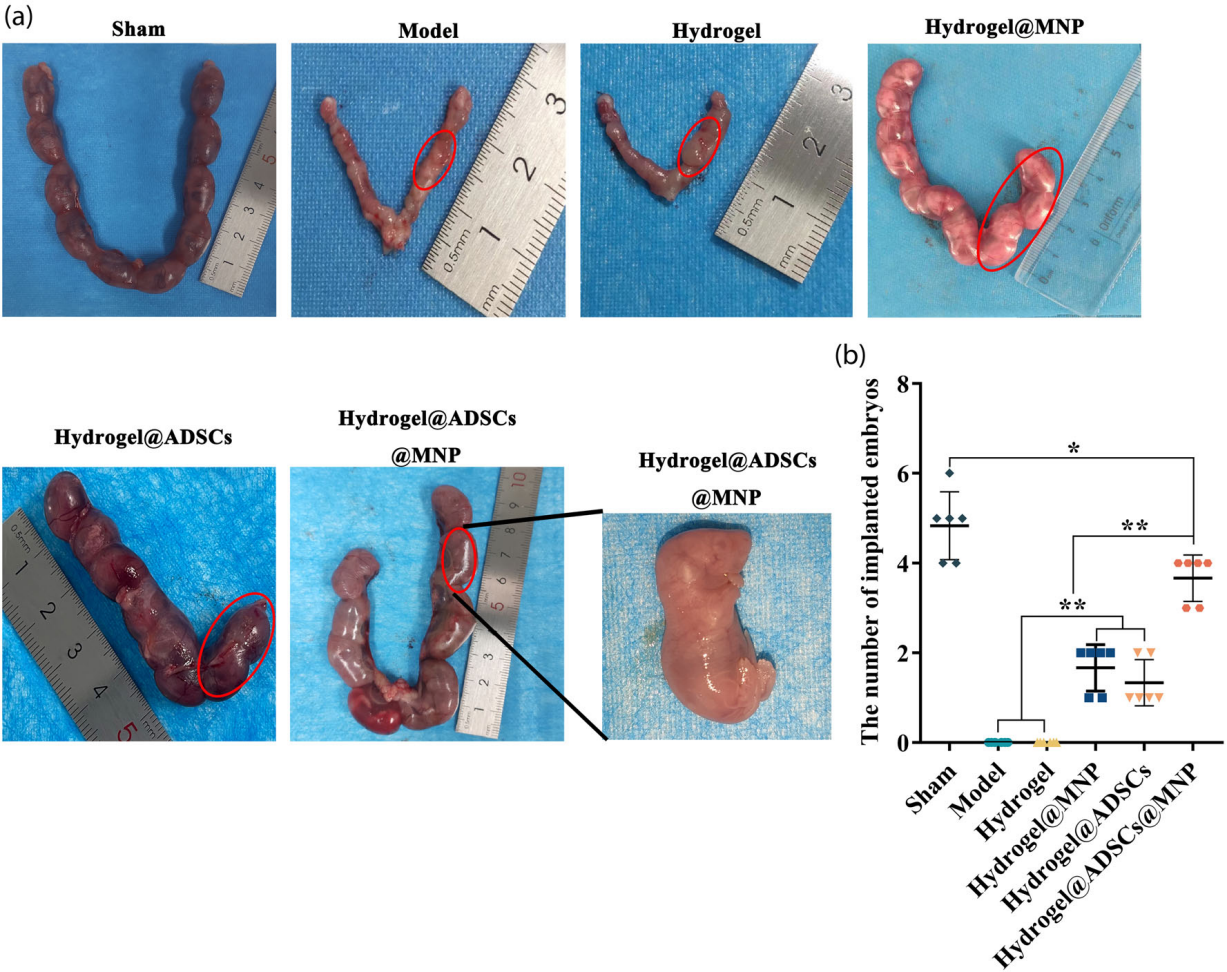
The influence of hydrogel@ADSCs@MNP treatments on fertility restoration. (a) Pregnancies in the different treatment groups (sham, model, hydrogel, hydrogel@MNP hydrogel@ADSC, hydrogel@ADSC@MNP). (b) Statistical examination of the number of implanted embryos in the scarred areas per uterine horn. *p < 0.05, **p < 0.01.

To investigate the impact of hydrogel@ADSC@MNP transplantation on the fertility of mice with endometrial injury. Subsequently, female and male mice were cohabited in cages after three estrous cycles. Implantation rates were assessed in each experimental group by collecting embryo. The embryo from hydrogel@ADSC@MNP group exhibited normal development (Figure 7a). As depicted in Figure 7a,b, the sham operation group exhibited the highest implantation number. In comparison with the model group, the implantation number in the hydrogel@ADSC@MNP‐treated group was significantly elevated (Figure 7b).

## 3 DISCUSSION

The basal layer of the endometrium is permanent and primarily functions through the activity of endometrial adult stem cells and progenitor cells, supplying cells for the generation of a new functional layer in each menstrual cycle.^25^ Although some studies have reported the use of mesenchymal stem cells for treating endometrial injury, the efficacy of such treatments remains to be improved.^14^, ^26^, ^27^, ^28^ In our previous research, we discovered that melanin nanoparticle materials exhibit a favorable effect in clearing reactive oxygen species (ROS).^29^ In our current study, we demonstrated that the transplantation of hydrogel@ADSC@MNP promotes endometrial regeneration and enhances endometrial receptivity and fertility in mice with endometrial injury. These findings provide evidence that hydrogel@ADSC@MNP treatment may play a crucial role in restoring endometrial function.

The endometrium undergoes cyclical changes throughout the menstrual cycle. To a certain extent, the thickness of the endometrium serves as a reflective indicator of its functional status and can predict endometrial receptivity. Furthermore, the thickness of the endometrium is intricately linked to the degree of adhesion. The more extensive and severe the adhesion, the greater the damage inflicted upon the endometrium, resulting in diminished endometrial receptivity.^30^

To comprehensively evaluate the endometrial status, we conducted histological assessments using H&E and Masson staining to examine the recovery of endometrial tissue following treatment with hydrogel@ADSC@MNP. Our study demonstrated that the hydrogel@ADSC@MNP treatment significantly enhanced endometrial growth, increased the number of glands, and mitigated endometrial fibrosis in mice with endometrial injury. These findings align with previous investigations utilizing MSCs for the treatment of endometrial injury.^31^, ^32^, ^33^ The contribution of BMSCs to the functional recovery of the endometrium involves interactions with uterine parenchymal cells.^28^ Considering our results and existing studies, the local injection of hydrogel@ADSC@MNP holds promise for fostering the recovery and growth of injured endometrial tissue.

Given that endometrial regeneration relies on angiogenesis to supply appropriate oxygen and nutrients to regenerating tissue,^34^ our investigation aimed to ascertain the association between the hydrogel@ADSC@MNP and endometrial angiogenesis in vivo. CD31 serves as a crucial indicator for assessing neovascularization. Our Western blot and immunofluorescence results indicate a significant increase in the protein expression of CD31 with the treatment of hydrogel@ADSC@MNP. Furthermore, we assessed the expression of VEGFA, known for promoting neovascularization and endothelial cell proliferation, thereby optimizing blastocyst implantation through the mediation of vascular permeability.^35^ In our study, both the mRNA and protein expression of VEGFA in the hydrogel@ADSC@MNP group were upregulated compared with the model group. This finding aligns with the previous study, which demonstrated VEGF, TGF‐β, PDGF, ANG, and other angiogenic cytokines secreted by ADSCs can synergistically promote the angiogenic process and facilitate tissue regeneration.^36^, ^37^ ADSCs have paracrine activity and secrete a broad spectrum of bioactive molecules which contribute various therapeutic effects, including facilitating angiogenesis.^38^ Moreover, studies indicate the presence of ADSCs in the endometrial basal layer, with potential direct differentiation into CK19/CK7‐positive endometrial stromal cells, and the expression levels of CD31 and vimentin were also higher in the ADSC treatment group compared with those in the PBS treatment group.^33^, ^39^ Our results corroborate recent reports indicating that ADSCs has an angiogenic effect on tissue repair.

In this study, we also examined the proliferation and apoptosis of endometrial cells. Immunofluorescence staining for Ki67 and Cleaved‐Caspase3 revealed that, following treatment with hydrogel@ADSC@MNP, the proliferation of endometrial cells was significantly superior to that in the model group. Simultaneously, it alleviated cell apoptosis, consistent with previous reports indicating that mesenchymal stem cell treatment can promote the proliferation of endometrial cells, suppress apoptosis, thereby facilitate endometrial regeneration and repair.^40^

Endometrial receptivity stands out as a crucial factor influencing embryo implantation. When considering treatment measures for endometrial injury, the focus should not only be on reducing fibrosis but also on closely monitoring the impact on endometrial receptivity. Endometrial receptivity is orchestrated by a myriad of factors that synchronize intrauterine changes conducive to blastocyst implantation. Notably, LIF and IGF‐1 stand out as pivotal indicators of endometrial receptivity.^41^, ^42^ LIF mediation encompasses multiple processes preceding and during implantation, including uterine preparation, decidualization, blastocyst growth and development, embryo‐endometrial interaction, and trophoblast invasion.^43^ Our findings suggest that, At the mRNA level, treatment with hydrogel@ADSC@MNP demonstrated a substantial increase in the expression of both LIF and IGF‐1, which indicated the restoration of endometrial receptivity.

ROS are intimately linked to the uterine microenvironment, and an excess of ROS‐induced oxidative stress can directly impede endometrial receptivity.^44^ Our earlier research in acute kidney injury therapy studies revealed that melanin nanoparticles possess the capability to eliminate free radicals based on their inherent adsorption capacity.^29^ In this study, we observed a notable enhancement in endometrial regenerative capacity and receptivity when MNP were added to the treatment group, compared with the group treated solely with ADCSs. Consequently, this improvement ultimately contributed to an elevation in reproductive potential.

Enhancing fertility stands as the paramount objective of endometrial injury repair.^45^ To validate the efficacy of hydrogel@ADSC@MNP treatment, we conducted mating between male and female mice in each group 4 weeks post‐treatment, meticulously observing the subsequent pregnancy outcomes. Our data indicated a noteworthy increase in the number of implanted embryos in the hydrogel@ADSC@MNP group, signifying a potential enhancement in uterine function attributed to the treatment. Furthermore，in terms of safety, multiple studies have reported that the degradability of the PF‐127 hydrogel enable its ideal application in transplantation.^46^, ^47^ Moreover, Due to its excellent safety profile, PF‐127 is one of the United States Food and Drug Administration (USFDA)–approved thermosensitive biodegradable hydrogels for clinical application.^48^ In this study, the hydrogel provided served as a temporary carrier for cells and MNP, and the degradable hydrogel also improved the safety of subsequent fetal development. Regarding fetal effects, preliminary observations from dissected fetuses in this study showed no significant differences in their development compared with the control group. Additionally, our system also includes MNP and ADSCs. Our previous work reported that MNP has good biosafety, biocompatibility, biodegradability.^21^, ^29^ However, although animal models have been used in preclinical studies to evaluate the efficacy and safety of ADSCs therapies, there is no doubt that large randomized and controlled clinical trials are essential to reach a final safety recommendation.^49^

In summary, our study conclusively demonstrates that hydrogel@ADSC@MNP transplantation in mice with intrauterine injury not only facilitates the regeneration of the endometrium but also orchestrates the regulation of molecular markers pertinent to endometrial receptivity. This multifaceted impact translates into an improvement in fertility outcomes, presenting hydrogel@ADSC@MNP transplantation as a viable and promising strategy to ameliorate the consequences of endometrial injury.

## 4 CONCLUSIONS

This study has demonstrated the safety and efficacy of transplanting hydrogel@ADSC@MNP into the uterine cavity following endometrial injury. Through the collection of stem cells, preservation of their viability, and extension of their contact duration with the damaged endometrial site, reduction in the ROS level, a noteworthy enhancement in the capacity for endometrial proliferation and differentiation. These findings suggest a hopeful outlook for patients experiencing endometrial injury, particularly those for whom pharmacological interventions have proven ineffective.

## 5 MATERIALS AND METHODS

### 5.1 Animals

All experiments involving mice were conducted in compliance with the ethical standards set forth by the Ethics Committee of Shanxi Medical University (Protocol No. SYDL2019002). The study adhered to all relevant institutional and national regulations governing the ethical treatment and utilization of animals. ICR mice were procured from the Animal Center of Shanxi Medical University. The mice were accommodated in a controlled environment maintained at a temperature of 23 ± 3°C, with a relative humidity of 44 ± 2%, and subject to a 12‐h light–dark cycle. All animals were provided with ad libitum access to both food and water.

### 5.2 Isolation and identification of ADSCs

Inguinal adipose tissue was procured from female ICR mice aged 3–4 weeks. The adipose tissue was dissected and subjected to enzymatic digestion using a 0.01% collagenase type I solution for 30 min at 37°C. Immediately after digestion, the sample was centrifuged, and the precipitate was retained. Subsequently, the cell pellet was re‐suspended in DMEM/F‐12 (Hyclone) supplemented with 10% fetal bovine serum (FBS, Hyclone), 1% penicillin–streptomycin, and 2 mM glutamine (Hyclone). The cell suspension was then cultured in a T25 flask at 37°C with 5% CO_2_ and humidified incubator. To identify the cell surface markers of ADSCs, flow cytometric analysis was utilized. This included the assessment of specific markers such as CD90, CD73, CD105, CD34, and CD45. For the evaluation of adipogenic and osteogenic differentiation, ADSCs within passages 3 were seeded into six‐well plates containing DMEM/F12 supplemented with 10% FBS. When these cultures reached a confluence of approximately 90%, they were treated with osteogenic or adipogenic differentiation media (Cyagen) for a period of up to 3 weeks, adhering to the manufacturer's instructions. To confirm the successful differentiation into adipocytes and osteoblasts, the cultures were subjected to assessments for lipid accumulation via Oil Red O staining and for mineral content through Alizarin Red staining (Solarbio).

### 5.3 Preparation of plain water‐soluble MNP

Tyrosine‐derived synthetic melanin (20 mg) was first dissolved in 10 mL of 0.1 N NaOH aqueous solution under vigorous stirring. After dissolving, HCl aqueous solution (0.1 N) was swiftly dropped into the obtained basic melanin solution to adjust the pH to 7.0 under sonication with output power = 10 W for 1 min. A bright black melanin aqueous solution was obtained. The neutralized solution was further centrifuged with a centrifugal‐flter (Amicon centrifugal flter device, MWCO = 30 kDa) and washed with deionized water and repeated several times to remove the produced NaCl. The aqueous solvent was removed by freeze‐drying to obtain 15 mg black solid of plain water‐soluble MNP.

### 5.4 Detection of MNP release in hydrogel

Dissolve 400 μg of MNP in 1 mL of physiological saline, then gradient dilute it for use. Utilize a spectrophotometer to measure the absorbance values of different concentrations of MNP in physiological saline at 600 nm, record the data, and plot a standard curve. Dissolve 5 mg of MNP in 2 mL of 20% hydrogel, and then place it in a 37°C incubator to solidify. Subsequently, immerse it in physiological saline. Use a spectrophotometer to measure the absorbance values of physiological saline at 600 nm at 12, 24, 48, 72, 96, 120, 144, 168, and 192 h. Finally, calculate the concentration of MNP released from the hydrogel into the physiological saline at different time points at 37°C.

### 5.5 Transwell

When the growth density of ADSC cells reached 70%, they were starved by replacing the culture medium with 5% FBS for 24 h. After digestion, 200 μL of cell suspension was taken and added to small chambers coated with hydrogel. After 24 h of incubation in the culture chamber, the chambers were removed, fixed with 4% paraformaldehyde for 30 min, stained with 0.1% crystal violet for 10 min in the dark, and then observed and counted.

### 5.6 In vitro biocompatibility assessment

ADSCs cells were used to evaluate the biocompatibility of the hydrogel and MNP materials. Hydrogel samples were passed through a 0.22 μm cell filter. ADSC cells were cultured in a 96‐well plate until they reached 50% confluence. Add 20% PF‐127, 25% PF‐127, and 30% PF‐127 separately into the medium. Incubate for 1, 3, and 5 days. Then add CCK‐8 reagent, incubate for 2 h, and measure the absorbance at 450 nm using an ELISA reader to obtain the OD value. Evaluate the effect of different concentrations of PF‐127 on cell proliferation by comparing the OD values of different groups. Cultivate ADSC cells in a 96‐well plate until reaching 100% confluence. Add Hydrogel and Hydrogel@MNP mixture separately. Incubate for 1, 3, and 5 days. Then add CCK‐8 reagent and measure the OD value. The cell viability level was evaluated by comparing the percentage of OD values from different treatment groups with the ADSCs group.

### 5.7 Animal models and experimental design

In brief, 90 nine‐week‐old female ICR mice with an average weight of 25–30 g were randomly and equally divided into six groups: sham‐operated group, model group, hydrogel group, hydrogel@MNP group, hydrogel@ADSC group, and hydrogel@ADSC@MNP group (15 mice/group). Female mice were subjected to anesthesia through intraperitoneal injection of 60 mg/kg pentobarbital. In the experimental groups, the vaginal opening of the mice was flushed with PBS in the sham operation group and with 95% ethanol for a duration of 30 s. Subsequently, the uterus of the experimental groups was injected with 100 μL of various substances, including PBS in the normal group, hydrogel, ADSCs cells encapsulated hydrogel, ADCSs and MNP (5 mg/mL) encapsulated hydrogel. Following the experimental procedures, the mice were humanely euthanized, and their uterine tissues were excised and either sectioned or frozen at Day 21 during the diestrus stage of three estrus cycles.

### 5.8 Histology analysis

The uterine tissues collected from each experimental group were initially fixed in a 4% paraformaldehyde solution for a duration of 48 h, followed by a 12‐h dehydration process in 70% ethanol. Subsequently, the fixed specimens were embedded in paraffin wax and sectioned into 5 μm‐thick slices. These tissue sections were then subjected to deparaffinization using xylene and a sequential rehydration process involving 100%, 95%, 90%, 80%, 70%, and 50% alcohol, concluding with immersion in distilled water. Finally, the rehydrated tissue sections were stained with hematoxylin and eosin (H&E) and Masson's trichrome staining viewed using an inverted microscope. The thickness of the epidermal layer was calculated using Image Pro Plus (IPP) software. Images were analyzed using Image Pro Plus (IPP) software to measure the thickness of the endometrium, the number of endometrial glands and the percent of fibrosis area.

### 5.9 Immunofluorescence staining

The slides were blocked with 5% normal goat serum in phosphate buffered saline for 1 h at room temperature. The slides were incubated with anti‐Vimentin (bs‐0756R; BIOSS), anti‐CD31 (BS‐0468R; BIOSS), anti‐CK18 (GB11232; Servicebio) anti‐Ki67 (E‐AB‐22027; Elabscience), anti‐VEGFA (19003–1; Proteintech), and anti‐Cleaved Caspase3 (9664; CST) antibodies overnight at 4°C. Secondary antibodies were added to the slides for 1 h at room temperature. The nuclei were counterstained with DAPI. Photographs were taken under a confocal microscopy.

### 5.10 Quantitative real‐time PCR

Total RNA was isolated from the porcine back skin tissues with Trizol (Invitrogen). Reverse transcription was performed using a FastQuant RT Kit (Tiangen Biotech). Quantitative real‐time PCR analyses were performed using TaKaRa SYBR Premix Ex Taq (TaKaRa, Tokyo, Japan) on an qTOWER 2.0 system (Analytikjena). The housekeeping gene *ACTB* was used as the internal control. The primer sequences are shown in Table S1. The relative expression of each gene was calculated using the comparative cycle threshold ($2^{-∆∆C_{t}}$) method. Statistical analysis was performed using GraphPad Prism 6.0. Differences with *p* < 0.05 were considered significant.

### 5.11 Western bolt analysis

The uterine tissues were meticulously dissected, rapidly frozen in liquid nitrogen, and subsequently preserved at a −80°C until they were required for further analysis. The uterus was homogenized in a RIPA lysis buffer. The proteins were subjected to SDS‐PAGE (Sodium Dodecyl Sulfate‐Polyacrylamide Gel Electrophoresis), followed by the transfer of proteins onto PVDF (Polyvinylidene Difluoride) membranes. To prevent non‐specific binding, the PVDF membranes were incubated in a 5% skim milk solution for 1 h. The primary antibodies, anti‐VEGFA (ab1316; abcam), anti‐Vimentin (bs‐0756R; BIOSS), anti‐CD31 (BS‐0468R; BIOSS), anti‐CK18 (GB11232; Servicebio) anti‐Ki67 (E‐AB‐22027; Elabscience) and anti‐Cleaved Caspase3 (9664; CST) and anti‐β‐actin (BM0627, 1:2000; Boster), were incubated with the blot membranes overnight at 4°C. The membranes were incubated with secondary antibodies for 1 h at room temperature. ECL Prime (GE Healthcare) was used to detect signals on the membrane.

### 5.12 Measurement of intracellular ROS levels

The ROS content was assayed using the commercial biochemical reagent kits (BB‐470538; Bestbio and) according to the manufacturer's protocols. The ROS level was quantified by fluorescence microplate reader (Bio‐Tek Instruments, USA) at the excitation wavelength of 488 nm and emission wavelength of 520 nm. The SOD activity was detected at a wavelength of 560 nm using a SOD assay Kit (BC0170, Solarbio, China) based on the xanthinoxidase method.

### 5.13 Fertility evaluation

A total of 36 mice were used in the fertility test. The fertility of each group of mice was assessed following three consecutive estrous cycles. Briefly, six female mice per group were allowed to mate naturally with male in cages, maintaining a female‐to‐male ratio of 2:1. At 16 days after the occurrence of vaginal suppository in female mice, the animals were euthanized to confirm pregnancy status.

### 5.14 Statistical analysis

Statistical data were expressed as mean ± SEM and at least three independent analyses. Categorical variables are expressed as number or percentage. Statistical significance was analyzed with an independent‐sample *t*‐test or two‐way ANOVA using GraphPad Prism software 6.0. Categorical variables (pregnancy rate) were compared using the Fisher's exact test. A value of *p <* 0.05 was considered statistically significant. *p < 0.05; **p < 0.01.

## AUTHOR CONTRIBUTIONS

**Ruigao Song:** Conceptualization; funding acquisition; methodology; writing – original draft. **Chicheng Ma:** Formal analysis; investigation; methodology; writing – original draft. **Hongxia Li:** Investigation; methodology; validation. **Yu Cheng:** Formal analysis; investigation. **Xianmei Cui:** Investigation; validation. **Zanhong Wang:** Methodology. **Lijuan Huang:** Resources. **Chunying Song:** Investigation; resources. **Yukai Jing:** Methodology. **Bing Cao:** Investigation. **Lili Wang:** Investigation. **Qing Tian:** Conceptualization; investigation. **Xi Wang:** Conceptualization; supervision. **Ruiping Zhang:** Conceptualization; resources; writing – review and editing. **Hanwang Zhang:** Conceptualization; methodology; project administration; writing – review and editing.

## CONFLICT OF INTEREST STATEMENT

The authors declare that they have no conflict of interest.

### PEER REVIEW

The peer review history for this article is available at https://www.webofscience.com/api/gateway/wos/peer-review/10.1002/btm2.10714.

## Supporting information


Table S1

## Data Availability

Some or all data and models generated or used during the study are available from the corresponding author by request.
