# Supplementary material for: A temperature responsive hydrogel encapsulated with adipose‐derived stem cells and melanin promotes repair and regeneration of endometrial injury
Source: Bioeng Transl Med. 2024 Aug 16;10(1):e10714. doi: 10.1002/btm2.10714 (PMC11711210; doi:10.1002/btm2.10714)
Supplement: Supplementary file 1 — Table S1 [file BTM2-10-e10714-s001.docx]

Supplemental material

Table S1. Oligonucleotides for real-time PCR

| Primer name | Sequence |
| --- | --- |
| VEGFA-RT-1F | GCTGCTGTAACGATGAAGCC |
| VEGFA-RT-1R | TTGACCCTTTCCCTTTCCTCG |
| FGF2-RT-1F | GGCTGCTGGCTTCTAAGTGT |
| FGF2-RT-1R | CCCAGTTCGTTTCAGTGCCA |
| IGF-1-RT-1F | CCTCAGACAGGCATTGTGGA |
| IGF-1-RT-1R | TGTTTGTCGATAGGGACGGG |
| Vimentin-1-RT-1F | GCAGTATGAAAGCGTGGCTG |
| Vimentin-1-RT-1R | ACCTGTCTCCGGTACTCGTT |
| LIF-RT-1F | ATCGGATGGTCGCATACCTG |
| LIF-RT-1R | CGGTACTTGTTGCACAGACG |
